# Simultaneous Semi-Mechanistic Population Pharmacokinetic Modeling Analysis of Enalapril and Enalaprilat Serum and Urine Concentrations From Child Appropriate Orodispersible Minitablets

**DOI:** 10.3389/fped.2019.00281

**Published:** 2019-07-09

**Authors:** Muhammad Faisal, Willi Cawello, Bjoern B. Burckhardt, Jan de Hoon, Stephanie Laer, LENA Consortium

**Affiliations:** ^1^Institute of Clinical Pharmacy and Pharmacotherapy, Heinrich-Heine-University Düsseldorf, Düsseldorf, Germany; ^2^Center for Clinical Pharmacology, University Hospitals Leuven/KU Leuven, Leuven, Belgium

**Keywords:** enalapril, enalaprilat, heart failure, population pharmacokinetics modeling analysis, NONMEM, orodispersible mini-tablets, child appropriate dosage forms

## Abstract

Enalapril is recommended as the first line of therapy and is proven to improve survival rates for treatment of Pediatric Heart Failure; however, an approved drug and child appropriate dosage formulation is still absent. The present analysis was conducted to perform a detailed model informed population pharmacokinetic analysis of prodrug enalapril and its active metabolite enalaprilat in serum and urine. Further, a model informed dosage form population-pharmacokinetic analysis was conducted to evaluate differences in pharmacokinetics of enalapril and its active metabolite enalaprilat when prodrug was administered to 24 healthy adults in a crossover, two periods, two treatments, phase I clinical trial using child-appropriate orodispersible mini-tablets (ODMT) and reference (Renitec®) dosage formulation. A simultaneous semi-mechanistic population-pharmacokinetic model was developed using NONMEM software, which predicted full profile serum and urine concentrations of enalapril and enalaprilat. First-order conditional estimation with interaction was used for parameter estimation. Transit compartments added using Erlang distribution method to predicted enalapril absorption and enalaprilat formation phases. Normalized body weight was identified as covariate related to enalapril volume of distribution. Visual predictive check (VPC) plots and conducted bootstrap analysis validated the model. The data from the two formulations were pooled for population-pharmacokinetic analysis and covariate effect of the formulation was found on mean transit time (MTT1) of enalapril absorption. In addition, data of each formulation were modeled separately and the estimated parameters of each individual administered both formulations were correlated using paired samples Wilcoxon rank test (*p* < 0.05 = significant) which also showed only a significant difference (*p* = 0.03) in MTT1 i.e., 5 min early appearance of enalapril from ODMT compared to reference tablets. No difference in the pharmacokinetics of active enalaprilat was found from the ODMT compared to the reference formulation. The population pharmacokinetic analysis provided detailed information about the pharmacokinetics of enalapril and enalaprilat, which showed that the ODMT formulation might have similar pharmacodynamic response compared to the reference formulation.

## Introduction

Despite the major success of the US Food and Drug Authority (FDA) and the European Medicine Agency (EMA) legislative and incentive initiatives toward pediatric drug development, the treatment of congestive heart failure in pediatrics (CHF) still lacks an approved drug and dosage formulation (1, 2). Lack of approved dosage forms leads to manipulation, modification, and extemporaneous administration of drugs (3, 4). These sub-optimal practices can result in compromised safety and efficacy and emphasize the need to develop a child appropriate dosage formulations (5, 6).

Enalapril has been recommended for the treatment of adult heart failure (7) and has been shown to prolong the survival rates in CHF patients (8). Around 60% of the administered oral dose of the drug is absorbed through the gastrointestinal tract. Enalapril is an inactive prodrug which is biotransformed in the liver by the carboxylesterases I (CES I) enzyme into an active angiotensin-converting enzyme inhibitor (ACE-I) enalaprilat (9, 10). Enalapril and enalaprilat are eliminated through the renal route without further metabolism. Around 60% of the administered dose is recovered in urine as enalapril and enalaprilat (11). At present, no other route of elimination has been reported for the enalapril and enalaprilat.

The ethical constraints in conducting pediatric clinical trials have restricted detail biopharmaceutical analysis of drugs and dosage formulations in children. Despite the physiological, biochemical, and pathological differences with pediatrics (12–14), healthy adult volunteers are usually hired and a non-compartmental analysis is conducted to assess the bioavailability and bioequivalence of drugs administered using child-appropriate dosage formulations (15).

One such initiative was taken by European commission to develop a child appropriate dosage formulation of enalapril for the treatment of CHF (16). A non-compartment analysis has been conducted to compare bioavailability and bioequivalence of enalapril administered using child-appropriate orodispersible mini-tablets (ODMT) with reference (Renitec®) tablet formulation (16). In addition, a model-dependent pharmacokinetic analysis has been conducted using least square minimization method of parameter estimation, which provided additional pharmacokinetics information including the same rate constant of absorption but an early appearance of enalapril from ODMT formulation compared to reference tablet formulation (8). Early drug absorption of active parent drugs from ODMTs may be useful in achieving an early pharmacodynamic response of some drugs especially those BCS Class I drugs which have a concentration-dependent pharmacodynamic response (17). However, for the inactive drugs like enalapril having a metabolite concentration-dependent pharmacodynamic response, the pharmacodynamic effect is expected to be dependent on the biotransformation rate and appearance time of metabolite in serum (18).

Therefore, the present analysis was conducted in which the data from the ODMT and reference formulation were pooled and the simultaneous semi-mechanistic population pharmacokinetic model was developed to predict the serum and urine concentrations and pharmacokinetics of the inactive prodrug enalapril. Simultaneously, the concentrations and pharmacokinetics of enalaprilat i.e., biotransformation of the prodrug from administered formulations to active metabolite and its disposition were predicted. The covariate effect of formulation on each population pharmacokinetic model estimated parameter was assessed. In addition to that, the data sets of each formulation were modeled separately and the estimated individual pharmacokinetic parameters of the drug and metabolite from the two formulations were statistically compared to account any pharmacokinetic differences.

The population pharmacokinetic modeling analysis was performed to obtain further deep evaluation and any differences in the pharmacokinetics of enalapril and bio-transformed active ACE inhibitor enalaprilat from the two formulations. This shall provide deeper insights regarding the expected pharmacodynamic effects of enalaprilat from the developed child appropriate ODMT formulation compared to the reference formulation.

## Method

### Study Design

The dataset for the simultaneous serum and urine population pharmacokinetic modeling analysis consisted of full time vs. serum and urine concentration profiles of enalapril and its active metabolite enalaprilat. The dataset was obtained from a two-phase, two treatment, crossover, phase I clinical trial, which was the part of LENA project (labeling of enalapril from neonate to adolescence, European Union Seventh Framework Program (FP7/2007-2013) under the grant agreement no 602295). The independent Ethics Committee of the University Hospitals KU Leuven approved the trial protocol and the study was conducted in accordance with the International Council on Harmonization (ICH) tripartite Guideline on Good Clinical Practices (19). The design of the clinical study has been publically outlined (16). In this study, 10 mg single extravascular dose of enalapril maleate was administered in two different periods to 24 healthy adult volunteers to compare the pharmacokinetics of drug and metabolite from the two formulations. Reference treatment consisted of two 5 mg strength of enalapril maleate market authorized conventional tablet formulation (Renitec®) administered with 240 ml of water. The ODMTs consisted of 10 child appropriate mini-tablets of 1 mg strength administered simultaneously with 240 ml of water. Serum samples were collected at the time intervals of 0.17, 0.33, 0.5, 0.75, 1, 1.25, 1.5, 2, 2.5, 3, 3.5, 4, 4.5, 5, 6, 8, 10, 12, 24, 48 h after dose administration. Enalapril and enalaprilat urine samples were collected at the time intervals of, 0–2, 2–4, 4–8, 8–12, 12–24, 24–36, 36–48 h after the dose administration. The urine concentrations were converted into the cumulative amount of the drug and metabolite excreted in urine after each time interval. The data sets of drug and metabolite concentrations were merged into a single data set for a simultaneous population pharmacokinetic modeling analysis.

Information related to biometric covariates including age, weight, height, sex, and total body water was available to evaluate their relationship with model parameters (Table 2). The total body water parameter was estimated for male and female subjects by Equations 1 and 2 (20).

Total body water for male subjects was calculated by using Equation 1;

(1) $$\begin{array}{l} Total\text{ body water }Vd\;\left(L\right)=0.3625\;*\text{ Body Weight }\left(\text{kg}\right)\\ +0.2239\;*\text{ Height }\left(\text{cm}\right)-0.1387\;*\text{ Age }\left(\text{yr}\right)-14.47 \end{array}$$

Total body water for female subjects was calculated by using Equation 2;

(2) $$\begin{array}{l} Total\;body\;water\;Vd\;\left(L\right)=0.2363\;*\text{ Body Weight }\left(\text{kg}\right)\\ +0.1962\;*\text{ Height }\left(\text{cm}\right)-0.0272\;*\text{ Age }\left(\text{yr}\right)-10.26 \end{array}$$

### Bioanalysis of Serum and Urine Samples

For a reliable determination of unknown samples, 50-μL serum or 100-μL urine were necessary for the simultaneous determination of enalapril and enalaprilat. In-house solid-phase extraction protocols were applied to purify serum and urine samples before liquid chromatography coupled to tandem mass spectrometric analysis. While a simple protocol was sufficient for serum samples (MAX 96-well plate), the known high intra and inter-subject variability of urine samples could only be sufficiently controlled by a two-step cleaning approach applying WAX followed by MCX solid phase extraction. The eluates were evaporated to dryness under a gentle stream of nitrogen. The established protocols enabled process efficiencies of the urinary solid-phase extraction between 87.2 to 106.8% (enalapril) and 64.1 to 90.2% (enalaprilat) (put a reference from literature here/see below). The serum process efficiency ranged between 65 to 93% for enalapril and 95 to 119% for enalaprilat.

Chromatographic separation and detection for both biological matrices were performed on a modular Shimadzu HPLC 10 coupled with AB Sciex API 2000 mass spectrometer. The ion transitions were the mass-to-charge ratio (m/z) 377.3 to 234.2 m/z for enalapril, 349.3 to 206.1 m/z for enalaprilat, and 425.3 to 351.2 m/z for benazepril (IS). The data acquisition and processing were carried out with Analyst 1.5.1 (Applied Biosystems/MDS SCIEX, Concord, Canada) with IntelliQuan® as an integration algorithm without smoothing.

Concerning the determination in serum, the fully validated bioanalytical method was characterized by a small sample volume of 50-μL serum encompassing a calibration range from 0.195 to 200 ng/mL for enalapril and 0.180 to 188 ng/mL for enalaprilat. Obtained mean accuracy values ranged from 91.6 to 108.4% of the nominal concentrations for enalapril and from 88.0 to 106.4% for enalaprilat. The time-different intermediate precision for the drug substance enalapril varied between 5.0 and 9.5% across all concentration levels and was subsequently well within the guideline limits of ±15% (±20% at LLOQ). The same applies to enalaprilat (4.3 to 13.4%). The relative matrix effect (expressed as CV) in enalapril samples at a low concentration (0.39 ng/mL [enalapril] and 0.35 ng/mL [enalaprilat]) was 5.49% for enalapril and 12.56% for enalaprilat. At the ULOQ, a coefficient of variation of 1.87% for enalapril and 8.96% for enalaprilat was evaluated for all seven different human sources.

The calibration curves of the urine method were constructed in the range of 11.6–1,2000 ng/mL for enalapril and 8.8–9,000 ng/mL for enalaprilat. The mean relative error across all *four* quality control levels ranged from −2.0 to 4.3% for enalapril and from −0.7 to 1.8% for enalaprilat. Intra-run and inter-run precision were 2.4–6.1 and 3.9–7.9% for enalapril as well as 3.1–9.4% and 4.7–12.7% regarding enalaprilat. The obtained variation coefficient of the IS-normalized matrix effects was 4.04 and 8.97% for enalapril and enalaprilat at the lower limit of quantification. At the upper limit of quantification, the CV was determined as 1.22% for enalapril and 1.21% for enalaprilat.

All unknown samples were quantified using freshly prepared calibration curves by plotting the concentration ratio of enalapril/enalaprilat to IS against the peak area of enalapril/enalaprilat to IS. Enalapril and enalaprilat were obtained as European Pharmacopeia Reference Standard. Study samples measured below the LLOQ were not included in the population pharmacokinetic modeling analysis (21).

### Population Pharmacokinetic Modeling Strategy

1. A simultaneous semi-mechanistic population pharmacokinetic model was developed to predict the pooled data of serum and urine concentrations of enalapril and its active metabolite enalaprilat representing the ODMTs and the reference formulations. Covariate analysis was conducted to test the effect of formulation on estimated model par ameters.
2. In addition to this, the data of ODMT and reference formulations were modeled separately and the individual model estimated pharmacokinetic parameters were statistically correlated to account any difference in the pharmacokinetics of drug and metabolite from the two formulations.

#### Population Pharmacokinetic Model Structure

Population pharmacokinetic model was developed using a non-linear mixed effect modeling software NONMEM version 7.4.0 (ICON, Development Solutions, Elliot City, MD, USA) (22). The ADVAN 6 sub-routine was used to define the system of differential equations whereby each compartment was connected by constants of first-order rate transfer. The one and two-compartment models were tested for enalapril. The one, two and three-compartment models were tested to predict enalaprilat concentrations in the combined model. Selection of the appropriate model was based on visual inspection of the goodness of fit plots (23), successful convergence, acceptable relative standard error values, a significant drop in the objective function value and no boundary problems. Maximum likelihood approach using first-order conditional estimate with interaction (FOCE+I) was used for parameter estimation.

After the analysis of goodness of fit plots, objective function value, and the estimated relative standard errors, the one-compartment model disposition parameters were selected for the population pharmacokinetic modeling of enalapril. Previous studies have reported that around 60% of the total administered enalapril is absorbed from the gastrointestinal tract (24, 25). Therefore, the bioavailability (F1) parameter was estimated to account the total amount of drug absorbed in central circulation. As per our current knowledge in the literature (25), we assumed that the enalapril and enalaprilat are only eliminated through urine and the drug is only metabolized to enalaprilat, which is not further metabolized. The assumption is supported by the reported value of the total amount of dose recovered in urine as enalapril and enalaprilat which is equal to the total amount of drug absorbed from the gastrointestinal tract (11). As we have serum and urine data for the drug and metabolite, the system becomes quantifiable and F1 parameter becomes identifiable and was estimated in the model. The estimated value of F1 parameter was 60% and was in line to already published value of 60% of drug absorption given in the literature (24, 25). To predict the lower concentrations at the delayed absorption phase of enalapril, a lag time model and system of transit compartments were used and analyzed (26). Transit compartments were added in a stepwise approach using erlang distribution method where the optimum number of transit compartments were estimated by adding one transit at a time until there was no further drop in the objective function value of 3.8 or more was observed (27, 28). The mean transit time parameter (MTT1) was estimated and rate transfer through transit compartments was calculated using the expression MTT1 = N+1/KTR, where N is the optimal number of transit compartments and KTR is the rate transfer through these transits (27). The rate constant of absorption (KA) was the transfer rate of the drug from the last transit into enalapril central compartment. To account the renal and metabolic elimination pathways, the elimination of enalapril from serum was estimated using rate constants of enalapril elimination through urine (KREN) and eliminated through enalaprilat formation (KM). The volume of distribution of enalapril in the central circulation (VC) and highly perfused tissues was estimated.

**Figure 1:**
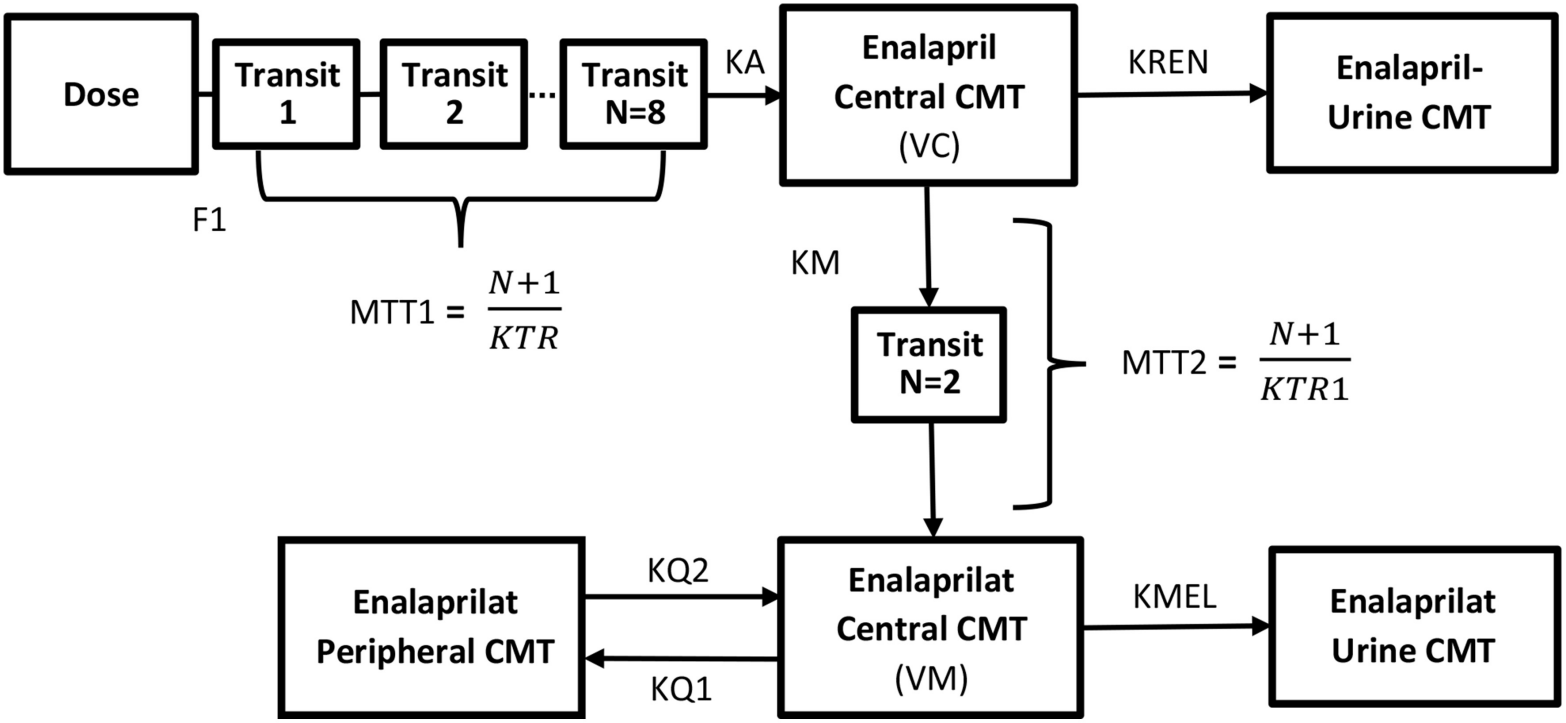
Blueprints of the semi-mechanistic population pharmacokinetic model of enalapril and enalaprilat in serum and urine after the administration of 10 mg of enalapril maleate using reference and ODMT formulations. The system of transit compartments with drug passing through by rate constant **(KTR)** was used to describe the absorption phase of enalapril, whereas the parameter **(F1)** subtracted the amount of drug metabolized pre-systemically in the gastrointestinal tract. The parameter **(KA)** describes the rate constant of absorption of the enalapril into the central compartment and instantaneously distribute to highly perfused tissues with a volume of distribution equals **(VC)**. The biphasic elimination of the drug i.e., bio-transformed to enalaprilat and eliminated via urine is described by the parameters **(KM)** and **(KREN)** from the central compartment. The formed metabolite transits through the delay compartments by mean residence time **(MTT2)** into the central compartment and distributing in the central **(VM)** and to the peripheral compartments **(KQ1 and KQ2)**. The elimination of enalaprilat takes place through the urine compartment **(KMEL)**.

The two-compartment model parameters estimated for the modeling of enalaprilat concentrations were rate constant of enalaprilat formation (KM), volume of distribution of enalaprilat in central circulation (VM), rate constants of intercompartmental distribution (KQ1 and KQ2), rate constant of elimination (KME) and mean transit time of enalaprilat formation (MTT2). Transit compartments were added using erlang distribution method to predict the lower concentrations enalaprilat formation phase. All the model parameters of enalapril and enalaprilat were identifiable. The blueprints of the combined model are given in Figure 1.

Between-subject variability of parameters was modeled using exponential error model described as Equation 3.

(3) Pi  =  TVp   ∗exp(ETAi)

Where **Pi** was the individual parameter estimate, **TVp** was the typical mean estimated value of the parameter of the population and **ETAi** was the individual random effect for each parameter per individual. The distribution of ETA in population was assumed to be following a normal distribution with mean zero and variance equals omega square (29).

Combined additive plus proportional residual error was introduced separately for serum concentrations and separate proportional error was introduced for urine concentrations of enalapril and enalaprilat, respectively, to account unexplained variability between the observed and predicted concentrations. Residual variability was defined using Equations 4 and 5.

(4) Ci=Cp   ∗(1+εi1)+ εi2

(5) Ci=Cp   ∗(1+εi1)

Where **C**_**p**_ was the predicted concentrations, **εi** represented the distribution of residuals between enalapril observed and model-predicted concentrations added by both proportional **εi1** and additive terms **εi2**. The distribution of residual variability was assumed to be normally distributed with mean zero and variance sigma squared.

#### Covariate Modeling Analysis

To build a full population pharmacokinetic model, a stepwise approach was used to evaluate the effect of estimated parameters on biometric covariates. The effect of normalized body weight added on the parameter estimates was evaluated using Equation 6 (30).

(6) $$TV=\theta {\;}_{TV}\;*\;{\left(\frac{WT_{ind}}{WT_{ref}}\right)}^{\theta }$$

Where **TV** indicates the typical population value of the model estimates, **θ_TV_** indicates the typical value of the model estimates for an individual; **WT**_**ind**_ indicates body weight of individual subject and **WT**_**ref**_ indicates weight normalized by the mean body weight of the present study. The parameter **θ** was tested with a fixed value of 1 for the volume of distribution and 0.75 for clearance. The inclusion of covariate was subjected to a significant drop in the objective function of more than 3.8.

For the pooled data analysis, the exponential relationship was incorporated using Equation 7 to evaluate the covariate effect of formulation on all model-estimated parameters.

(7) $$TV=\theta X*\theta 12^{FORM}*\exp^{\eta }$$

Where TV represents typical population value of model parameters, θ_X_ represents the mean population value of the model parameters, θ_12_ represents a fixed effect parameter to give a proportional increase or decrease in parameter value with ODMT (FORM = 1) or reference formulation (FORM = 0), and η represents interindividual variability.

#### Model Evaluation

**Figure 2:**
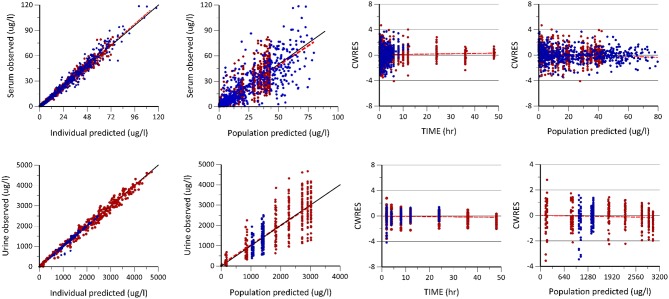
Goodness of fit plots including the observed vs. individual and population predicted plots, conditional weighted residuals (CWRES) vs. population predicted (PRED) and time plots of enalapril and enalaprilat in serum and urine generated after the population pharmacokinetic modeling of pooled data of two formulations. The first row shows the predictive performance of the model for enalapril and enalaprilat in serum. The second row shows the predictive performance of enalapril and enalaprilat in urine, respectively. The blue dots represent enalapril concentrations, whereas the red dots represent the enalaprilat concentrations in serum and urine, respectively.

The goodness of fit plots were used for the evaluation of model performance. For the evaluation of individual *post-hoc* estimation with FOCE+I method of parameter estimation method, conditional weighted residuals were estimated and visual plots given in Figure 2 were analyzed. Visual predictive check (VPC) plots and the non-parametric bootstrap method were used for model validation. For VPC plots, the final model was used to perform 1000 Monte Carlo simulations (31). The precision of the estimated model parameters was evaluated using a non-parametric bootstrap method using Perl-speaks-NONMEM (PsN) (32) by calculating the confidence interval (CI) of the estimated model parameters. The subjects of the original dataset were randomly resampled to create 200 datasets and the new dataset was modeled using the final model. From the bootstrap analysis, the 2.5th, 50th, and 97.5th percentiles were simulated. Table 1 summarizes the results of the bootstrap analysis with 95th percent CI values of each parameter.

**Table 1:** Enalapril and enalaprilat estimated population pharmacokinetic parameters with percent relative standard errors (RES), sampling importance resampling (SIR) and bootstrap confidence interval (CI) values estimated for pooled data analysis.

**Parameters**	**Population estimates (% RSE)**	**SIR 95th% CI**	**Bootstrap 95th% CI**
	**FINAL (FULL) MODEL**	**FINAL MODEL**	**FINAL MODEL**
**BASIC PHARMACOKINETIC MODEL PARAMETERS**			
KA (1/h)	6.010 (15.0%)	4.780–7.859	4.640–8.200
VC (L)	51.10 (4.00%)	47.92–54.34	47.38–55.09
F1	0.606 (3.00%)	0.580–0.640	0.570–0.646
MTT1 (h)	0.558 (9.00%)	0.480– 0.640	0.466–0.648
KREN (1/h)	0.305 (4.00%)	0.290–0.320	0.286–0.330
KM (1/h)	0.688 (4.00%)	0.640–0.740	0.647–0.725
VM (L)	46.10 (4.00%)	42.80–49.55	42.21–49.79
KQ1 (1/h)	0.060 (4.00%)	0.056–0.064	0.056–0.064
KQ2 (1/h)	0.054 (10.0%)	0.046–0.063	0.048–0.620
KME (1/h)	0.184 (4.00%)	0.171–0.196	0.171–0.197
MTT2 (h)	0.910 (8.00%)	0.800–1.044	0.802–1.080
THETA (X)	0.730 (12.0%)		
**INTERINDIVIDUAL VARIABILITY (IIV)**			
IIV_KA	0.688 (31.0%)	0.474–1.132	0.362–1.005
IIV_VC	0.058 (24.0%)	0.041–0.086	0.039–0.080
IIV_F1	0.041 (22.0%)	0.030–0.060	0.023–0.067
IIV_MTT1	0.151 (22.0%)	0.114–0.220	0.097–0.192
IIV_KREN	0.058 (24.0%)	0.042–0.084	0.038–0.074
IIV_KM	0.078 (22.0%)	0.058–0.112	0.050–1.101
IIV_VM	0.069 (23.0%)	0.050–0.102	0.033–0.105
IIV_KME	0.063 (23.0%)	0.047–0.092	0.039–0.086
IIV_ MTT2	0.296 (22.0%)	0.221–0.430	0.196–0.384
**RESIDUAL UNEXPLAINED VARIABILITY (RUV)**			
**Serum Enalapril**			
Proportional error (σ2)	0.010 (8.00%)	0.008–0.011	0.006–0.013
Additive error (μg/l)	0.188 (15.0%)	0.150–0.240	0.132–0.294
**Serum Enalaprilat**			
Proportional error (σ2)	0.018 (9.00%)	0.015–0.020	0.010–0.026
Additive error (μg/l)	0.220 (13.0%)	0.180–0.277	0.144–0.301
**Urine Enalapril**			
Proportional error (σ2)	0.019 (9.00%)	0.016–0.021	0.009–0.028
**Urine Enalaprilat**			
Proportional error (σ2)	0.005 (11.0%)	0.004–0.006	0.002–0.009

***KA**, rate constant of enalapril absorption; **VC**, Volume of distribution of enalapril in central compartment; **F1**, bioavailability of enalapril after extravascular administration; **MTT1**, delay time for enalapril to appear in serum; **KREN**, Rate constant of enalapril elimination in urine; **KM**, rate constant of metabolite formation; **KQ1**, Rate constant of enalaprilat distribution from central to peripheral compartment; **KQ2**, Rate constant of enalaprilat distribution from peripheral to central compartment; **KME**, rate constant of enalaprilat elimination in urine; **VM**, Volume of distribution of enalaprilat in urine; **MTT2**, delay time for enalaprilat to appear in serum*.

Due to the long run time of model, a faster than bootstrap method i.e., Sampling importance-resampling (SIR) method was also used to evaluate parameter uncertainty. SIR was performed by running 20,000 final samples and a resample size of 2,000. From SIR method, 95th, CI values were estimated for parameter uncertainty test (33, 34).

For the evaluation of the stability of estimated parameters of the model, initial fixed and random effect parameter values were changed stepwise by 10 percent and the population-estimated parameters along with the objective function value were assessed for any change.

#### Correlation of Reference and Child Appropriate Dosage Forms

The covariate effect of formulation on estimated model parameters of the pooled data was assessed using Equation 7. In addition to this, the estimated individual pharmacokinetic parameters of enalapril and enalaprilat of each subject administered ODMTs and reference formulations in two-phase crossover trial were statistically correlated using a paired sample Wilcoxon rank test using an R program with a significant level of *p* <0.05. The rate constants of enalapril and enalaprilat renal elimination were converted to their respective clearance values to correlate the clearances of drug and metabolite following the administration of both formulations.

## Results

The dataset of enalapril and the active metabolite enalaprilat consisted of 24 healthy subjects with 2,208 serum and urine concentrations. Patients' demographics are summarized in Table 2. After the extravascular administration, the C_max_ of enalapril was achieved in almost 1 h and time vs. serum concentration of enalapril showed a mono-exponential decay. The drug is eliminated in almost 10 h from the serum. Biotransformed enalaprilat achieved C_max_ in around 3 to 4 h and showed a bi-phasic exponential decay.

**Table 2:** Summary of biometric covariate values used in population pharmacokinetic analysis.

**Biometric covariate**	**Mean**	**Median**	**Minimum**	**Maximum**
AGE (yrs.)	28.00	24.40	22.08	47.16
Weight (kg)	69.76	67.60	51.80	95.60
Height (cm)	174.5	176.0	153.0	189.0
Body water (L)	42.06	41.31	32.86	53.70

### Model Evaluation Results

**Figure 3:**
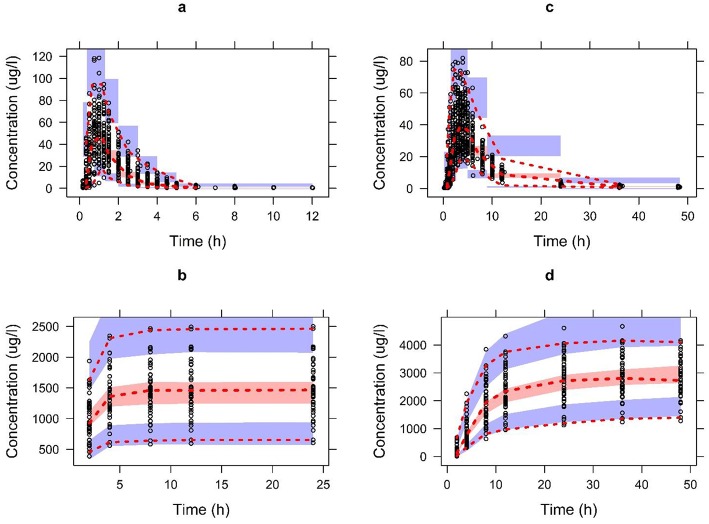
The visual predictive check plots for the final model of enalapril in serum **(A)** and urine **(B)** and enalaprilat in serum **(C)** and urine **(D)** validating the model performance to describe the pooled observed data. Open circles represent the pooled observed data of drug and metabolite. The red dashed line represents the 2.5th, 50th, and 97.5th percentiles of the pooled observed data. The shaded areas represent the 95% CI of the 2.5th, 50th, and 97.5th percentiles of predictions.

The Table 1 summarizes the final population pharmacokinetic model parameter estimates, relative standard errors (RES), SIR and 95th% Bootstrap confidence interval values of the pooled data of the two formulations. For the formulations modeled separately, the typical population and diagnostic parameters along with diagnostic plots are given in Supplementary Material. The RES, SIR, and bootstrap confidence intervals for all parameters were narrow and had the same median values as estimated by the model. This shows that parameters were precisely estimated. The goodness of fit plots given in Figure 2 shows that the model performed well in predicting the serum and urine concentrations of enalapril and enalaprilat. Eta shrinkage values for most of the parameters were lower than 20 percent and hence the individual model predicted concentration vs. the observed serum and urine concentration plots of enalapril and enalaprilat given in Figure 2 were informative and showed agreement between the observed and predicted concentrations (35). The VPC plots given in Figure 3 showed no model misspecification and almost all of the serum and urine observed concentrations were within the prediction intervals.

Visual inspection of the time vs. enalapril concentration plot showed a mono-exponential phase of elimination and did not have sufficient data for the estimation of the peripheral volume of distribution. The selected one compartment model adequately predicted the time vs. enalapril serum concentrations. The two-compartment model resulted in a significant drop in the objective function but at the expense of higher relative standard errors of the estimated parameters. An optimum number of transit compartments were added using erlang distribution method to account the absorption phase of enalapril. Eight transits (transits = 8) were added for reference and pooled data analysis and 6 transits were added for the ODMT formulation (transits = 6). The absorption phase of enalapril was not adequately predicted with or without incorporating a LAG time model. The introduction of transit compartments resulted in a significant drop in the objective function compared to the lag time model. The bioavailability parameter (F1) accounted for the percent of drug absorbed while subtracting it from the drug eliminated by pre-systemic metabolism. The enalapril population mean parameter estimates along with their relative standard errors are given in Table 1.

The biphasic enalaprilat time vs. serum concentration profile was predicted using the two-compartment model. The one compartment model was not able to predict the second longer phase of elimination. The two-compartment model also resulted in a significant drop in the objective function value compared to the one compartment model. A lag time parameter was not able to predict the lower concentrations at the skewed formation phase of enalaprilat; therefore, two-transit compartments were added using erlang distribution method to incorporate a delay in metabolite formation. First order rate of enalapril and enalaprilat elimination was adequate to predict the cumulative amount of drug and metabolite excreted in the urine. The structure explained in Figure 1 constituted the serum and urine simultaneous semi-mechanistic population pharmacokinetics of enalapril and enalaprilat. Structural population parameters and random variability values have been summarized in Table 1. The fixed effect parameters and the random variance was precisely estimated along-with the eta-shrinkage values, lower than 25%.

The forward addition of potential covariate i.e., weight normalized on the volume of distribution of enalapril resulted in a significant drop of the objective function value by 18.2, similarly, the addition of formulation effect on mean transit time (MTT1) of enalapril further resulted in a significant drop of the objective function by 6.51. The weight normalized on the volume of distribution was tested using the backward elimination method and resulted in a significant increase in the objective function value. Therefore, both covariates were retained in the final population model.

As like the base model, the relative standard error values of the fixed and random effect parameters of the final covariate model showed a precise estimation of the parameters. The change in the typical population and random variability of the parameters of the base and full covariate models were <25 percent and show that the covariates are clinically unimportant (35).

### Pharmacokinetics Comparison of ODMT and Reference Formulation

The pooled data analysis revealed that the formulation had a covariate effect on the mean transit time of enalapril absorption. Similarly, the results of the paired samples Wilcoxon rank test given in Table 3 showed a significant difference between the mean transits times of enalapril (MTT1) when the drug was absorbed from ODMT compared to conventional tablets. The typical population mean value of MTT1 showed that the drug was absorbed around 5 min earlier from ODMT compared to the reference formulation. No other pharmacokinetic differences in the comparison of the two formulations were observed. The statistical comparison showed that the two formulations are relatively bioavailable. The pharmacokinetic comparison showed that the drug and metabolite had a similar volume of distribution and clearance from the body.

**Table 3:** Result of the two-sided paired Wilcoxon rank sum test to compare pharmacokinetic parameters of enalapril and enalaprilat in serum and urine after the administration of enalapril using ODMT and reference formulation.

***P*-value**	**ODMT vs. Reference formulation**
KA (1/h)	0.87
VC (L)	0.85
F1	0.19
CLREN (L/h)	0.19
MTT1 (h)	0.03
KM (1/h)	0.70
VM (L)	0.13
MTT2 (h)	0.26
CLENT (L/h)	0.22

***KA**, rate constant of enalapril absorption; **VC**, Volume of distribution of enalapril in central compartment; **F1**, bioavailability of enalapril after extravascular administration; **CLREN**, Clarence of enalapril in urine; **MTT1**, delay time for enalapril to appear in serum; **KM**, rate constant of enalaprilat formation; **VM**, Volume of distribution of enalaprilat in central compartment; **MTT2**, delay time for enalaprilat to appear in serum; **CLENT**, Clarence of enalaprilat in urine*.

## Discussion

A Validated simultaneous semi-mechanistic population pharmacokinetic model adequately predicted the full profiles of serum and urine concentrations of enalapril and enalaprilat. A covariate analysis on population pharmacokinetic model parameters of pooled data showed the effect of formulation on the estimated mean transit time of enalapril absorption from the two formulations. In addition to the pooled data analysis, the statistical comparison of individual pharmacokinetic model parameters estimated separately for ODMTs and reference formulation data revealed that enalapril administered using ODMTs absorbed 5 min earlier than the reference tablet formulation. However, no difference in the rate and onset of the formation and disposition of the active ACE-I enalaprilat was noticed. Therefore, no differences in the pharmacodynamic effects are expected from ODMTs compared to the reference formulation.

Selection of the final model was based on the successful convergence with no boundaries, goodness of fit plots, acceptable relative standard errors, and a significant drop in objective function value. Visual predictive check plots (31), bootstrap analysis (32), SIR procedure (34) validated the model. The calculated relative standard errors showed that the model parameters were estimated with acceptable precision.

The model informed pharmacokinetic estimates given in Table 1 were in line to the already published value and showed that the population pharmacokinetic model estimated reliable pharmacokinetic parameters. For instance, the estimated fraction of enalapril absorbed was 60% and was in line with the literature value of 60% (24, 25). Similarly, the estimated values of the rate constant of absorption and delay in the appearance of enalapril in serum were 6.03 1/h and 0.56 h and were in line to the respective reported value of 6.4–12 1/h and 0.5 h (36). The value of the total rate constant of enalapril elimination through renal and metabolic route was estimated to be 0.93 1/h and was in line to the reported value of 0.94 1/h (36). The value of the rate constant of metabolite formation (KM) estimated using transit compartment model was 0.69 1/h and was in line to the reported value of 0.9 1/h estimated using the LAG time model (7). The estimated value of the rate constant of enalaprilat elimination was 0.175 1/h and was in line to the reported value of 0.14 1/h (36).

Semi-mechanistic population pharmacokinetic models have been reported in the literature to predict plasma and urine concentrations of drug and metabolite (37). A simultaneous enalapril and enalaprilat population pharmacokinetic model has not been reported in the literature. Based on the goodness of fit plots, objective function, and precision of parameters the selected one-compartment model was adequate to predict enalapril concentrations and has already been reported in the literature (8, 36). The two-compartment model used for enalapril estimated high standard errors of one or more parameters with no improvement in the goodness of fit plots and therefore was rejected (29). The one compartment model using the first order of absorption without accounting a delay in absorption did not account the absorption phase of enalapril. The LAG time model used to incorporate a delay in absorption did not predict the lower concentrations. A system of transit compartments was used to account the lower concentrations of the absorption phases of enalapril. The use of transit compartments also resulted in a significant drop in the objective function compared to the LAG time model. The transit compartments were added sequentially and have been used in literature to account the absorption phase of drugs (28, 38).

The selected two-compartment model for enalaprilat significantly dropped the objective function as compared to the one compartment model. The one compartment model was also not able to predict the bi-exponential elimination phase of the metabolite. The two-compartment population pharmacokinetic model with proportional residual error model has been reported in the literature to model enalaprilat concentrations, however, a combined additive plus proportional residual error model significantly dropped the objective function and was used in our final base model (7). A three-compartment model was also tested but resulted in higher standard errors with no significant change in the objective function and was rejected.

The covariate analysis of the pooled data of the two formulations found that formulations have a covariate effect on the mean transit time of enalapril in serum. The pairwise statistical comparison of model parameters estimated separately for ODMT and reference formulation data also showed a difference in mean transit time of enalapril absorption from the two formulations. Comparison of the absolute values of the mean transit time of absorption showed around 5 min early appearance of enalapril in serum from ODMTs compared to the reference formulation. The early appearance of enalapril may be due to the higher surface area of ODMTs provided for fast dissolution and disintegration rates of the developed tablets compared to the reference tablet formulation. The absorption and plasma concentration profile of an orally administered drug also depends on its transit time and absorbability in the gastrointestinal tract (39). The drug from the small-sized disintegrated and dissolved ODMTs are expected to be emptied earlier from the stomach to reach rapidly at the site of absorption in the intestine (40). This may lead to the early availability of the drug for absorption. Enalapril is a BCS class III drug and follows a permeability-limited absorption from the intestine. Therefore, an early appearance of the drug had no effect on the rate constant of enalapril absorption.

The difference in the model informed transit time of enalapril in the gastrointestinal tract and different excipients of the reference and ODMT formulations had no effect on intestinal permeation i.e., rate constant of absorption of the drug. These results support the biowaivers given by the FDA to BCS class III drugs whereby the excipients should not have an effect on bioavailability and drug permeability (41, 42). No other differences in the physiological parameters like the volume of distribution and elimination were observed for enalapril. The implication of early appearance of the drug from ODMTs can be useful for classes of drugs like analgesics or in case of an emergency clinical situation such as a hypertensive crisis or an angina attack. For instance, fast disintegrating and dissolving small sized ODMT formulation of nitroglycerin if develop requires less saliva and can be more beneficial to deliver drug sublingually for achieving early antianginal effect compared to sublingual dosage forms which require more saliva and higher disintegration time to release the drug (43). Use of ODMTs may have earlier pharmacodynamic effects, especially for the orally administered BCS class I drugs having higher solubility and permeability. However, in our case, the pro-drug enalapril is inactive and its early appearance will have no expected clinical significance because the pharmacodynamic response will depend on the pharmacokinetics of enalaprilat.

The developed population pharmacokinetic model also informed that the early appearance of drug in the serum had no effect on the onset and rate of enalaprilat biotransformation. This may be due to the same rate constant of absorption and extent of absorption of enalapril from the two formulations. In addition, the uptake of the drug from the systemic circulation into the liver by organic anion-transporting polypeptide (OATP1B1) transporters (44) and the basolateral efflux of the bio-transformed enalaprilat back into the systemic circulation by multidrug resistance-associated protein (MRP4) transporters follows a permeability-limited transport (45). The volume of distribution and clearance of enalaprilat also showed no difference between the two formulations. Due to similar enalaprilat pharmacokinetics, a similar pharmacodynamic response can be expected from the reference and ODMT formulation.

Typically a non-compartmental analysis is conducted to compare the pharmacokinetic parameters of drug including area under the curve, maximum concentration in biological fluid, and time to get the maximum concentration in biological fluid from reference and developed formulations. However, the population pharmacokinetic modeling analysis has provided deeper insights relating to the onset of absorption and metabolism. The model can further be used to perform simulations in order to predict the serum concentrations of enalapril and enalaprilat at different dose and dosing frequencies.

## Conclusion

The semi mechanistic population pharmacokinetic model predicted the detailed pharmacokinetics of enalapril and enalaprilat in serum and urine. The model informs that enalapril is absorbed 5 min earlier when administered using ODMT compared to the reference formulation. The model also informed no difference in the pharmacokinetics of enalaprilat bio-transformed from the administered parent drug formulations. This may lead to a similar pharmacodynamic response from ODMTs and reference formulations.

## Data Availability

The datasets for this study will not be made publicly available because, the dataset is currently being used for further analysis and hence cannot be shared.

## Ethics Statement

Approval for the trial protocol was given by the independent Ethics Committee of the University Hospitals KU Leuven and the study was conducted in accordance with the international Council on Harmonization (ICH) Tripartite Guideline on Good Clinical Practice.

## Author Contributions

MF, WC, and SL contributed to the idea. MF and WC develop the overall population pharmacokinetic model. MF performed population pharmacokinetic modeling analysis. BB performed bioanalysis of plasma and urine samples. MF, BB, and WC contributed to the drafting of the manuscript. WC, SL, and JdH critically checked the manuscript. All authors agreed on the contents of this manuscript.

### Conflict of Interest Statement

The authors declare that the research was conducted in the absence of any commercial or financial relationships that could be construed as a potential conflict of interest.
