# Supplementary material for: Simultaneous Semi-Mechanistic Population Pharmacokinetic Modeling Analysis of Enalapril and Enalaprilat Serum and Urine Concentrations From Child Appropriate Orodispersible Minitablets
Source: Front Pediatr. 2019 Jul 9;7:281. doi: 10.3389/fped.2019.00281 (PMC6629781; doi:10.3389/fped.2019.00281)
Supplement: Supplementary file 1 [file Data_Sheet_1.docx]

**SUPPLEMENTARY MATERIALS:**

**Table Supp-I:** Serum and urine estimated population pharmacokinetic parameters, sampling importance resampling (SIR) and bootstrap confidence interval (CI) values of enalapril and enalaprilat from the **reference** f0ormulation.

**Table Supp-II:** Serum and urine estimated population pharmacokinetic parameters, sampling importance resampling (SIR) and bootstrap confidence interval (CI) values of enalapril and enalaprilat from the **ODMT** formulation

**Fig Supp-1:** Goodness of fit plots including the observed versus individual and population predicted and conditional weighted residuals (CWRES) versus population predicted (PRED) plots of enalapril and enalaprilat in serum and urine generated after the population pharmacokinetic modeling of **reference** formulation. The first and third row shows the predictive performance of the model for enalapril in serum and urine respectively. The second and fourth line shows the predictive performance of enalaprilat in serum and urine respectively.

**Fig Supp-2:** Goodness of fit plots including the observed versus individual and population predicted and conditional weighted residuals (CWRES) versus population predicted (PRED) plots of enalapril and enalaprilat in serum and urine generated after the population pharmacokinetic modeling of **ODMT** formulation. The first and third row shows the predictive performance of the model for enalapril in serum and urine respectively. The second and fourth row shows the predictive performance of enalaprilat in serum and urine respectively.

| **Parameters** | **Population Estimates (% RSE)** | **SIR 95th % CI** | **Bootstrap 95th % CI** |
| --- | --- | --- | --- |
|  | **FINAL (FULL) MODEL** | **FINAL MODEL** | **FINAL MODEL** |
| ***Basic pharmacokinetic model parameters*** | | | |
| KA (1/h) | 7.780 (37.0 %) | 5.640-11.90 | 5.541-12.10 |
| VC (L) | 51.60 (6.00 %) | 46.31-57.06 | 45.00-55.40 |
| F1 | 0.630 (5.00 %) | 0.574-0.681 | 0.562-0.667 |
| MTT1 (hr) | 0.570 (10.00 %) | 0.492-0.681 | 0.475-0.643 |
| KREN (1/h) | 0.312 (5.00 %) | 0.281-0.341 | 0.274-0.339 |
| KM (1/h) | 0.683 (7.00 %) | 0.605-0.758 | 0.635-0.746 |
| VM (L) | 44.70 (5.00 %) | 40.98-48.87 | 42.10-50.78 |
| KQ1 (1/h) | 0.060 (6.0 %) | 0.054-0.065 | 0.053-0.068 |
| KQ2 (1/h) | 0.057 (15.0 %) | 0.046-0.071 | 0.045-0.067 |
| KME (1/h) | 0.192 (6.00 %) | 0.176-0.210 | 0.175-0.210 |
| MTT2 (h) | 0.942 (13.0 %) | 0.745-1.144 | 0.741-1.025 |
| ***Interindividual variability (IIV)*** | | | |
| IIV_KA | 1.310 (53.0 %) | 0.796-1.604 | 0.799-1.522 |
| IIV_VC | 0.069 (34.0 %) | 0.044-0.120 | 0.049-0.090 |
| IIV_F1 | 0.057 (31.0 %) | 0.039-0.089 | 0.041-0.750 |
| IIV_MTT1 | 0.203 (31.0 %) | 0.132-0.338 | 0.137-0.366 |
| IIV_KREN | 0.056 (33.0 %) | 0.036-0.097 | 0.035-0.085 |
| IIV_KM | 0.088 (32.0 %) | 0.056-0.141 | 0.050-0.134 |
| IIV_VM | 0.048 (35.0 %) | 0.029-0.081 | 0.036-0.102 |
| IIV_KME | 0.053 (33.0 %) | 0.033-0.088 | 0.035-0.086 |
| IIV_ MTT2 | 0.330 (32.0 %) | 0.225-0.420 | 0.243-0.350 |
| ***Residual unexplained variability (RUV)*** | | | |
| ***Serum Enalapril*** | | | |
| Proportional error (σ2) | 0.010 (12.0 %) | 0.007-0.011 | 0.006-0.014 |
| Additive error (ug/l) | 0.189 (21.0 %) | 0.137-0.269 | 0.125-0.245 |
| ***Serum Enalaprilat*** | | | |
| Proportional error (σ2) | 0.021 (14.0 %) | 0.016-0.026 | 0.014-0.026 |
| Additive error (ug/l) | 0.220 (22.0 %) | 0.159-0.310 | 0.147-0.316 |
| ***Urine Enalapril*** | | | |
| Proportional error (σ2) | 0.011 (14.0 %) | 0.009-0.014 | 0.007-0.016 |
| ***Urine Enalaprilat*** | | | |
| Proportional error (σ2) | 0.005 (16.0 %) | 0.004-0.006 | 0.003-0.009 |

**Table Supp-I:**

**Table Supp-II:**

| **Parameters** | **Population estimates**  **(% RES)** | **SIR 95^th^ % CI** | **Bootstrap 95^th^ % CI** |
| --- | --- | --- | --- |
|  | **FINAL (FULL) MODEL** | **FINAL MODEL** | **FINAL MODEL** |
| ***Basic pharmacokinetic model parameters*** | | |  |
| KA (1/h) | 7.71 (26.0 %) | 5.623-11.59 | 5.329-12.22 |
| VC (L) | 50.70 (5.00 %) | 46.87-55.05 | 47.00-55.59 |
| F1 | 0.589 (4.00 %) | 0.556-0.624 | 0.553-0.627 |
| MTT1 (hr) | 0.484 (7.00 %) | 0.438-0.551 | 0.417-0.553 |
| KREN (1/h) | 0.298 (5.00 %) | 0.274-0.323 | 0.274-0.325 |
| KM (1/h) | 0.693 (6.00 %) | 0.631-0.757 | 0.641-0.750 |
| VM (L) | 47.60 (7.00 %) | 42.52-53.20 | 42.65-52.93 |
| KQ1 (1/h) | 0.060 (5.00 %) | 0.056-0.066 | 0.054-0.066 |
| KQ2 (1/h) | 0.051 (14.0 %) | 0.040-0.064 | 0.042-0.060 |
| KME (1/h) | 0.175 (6.00 %) | 0.158-0.193 | 0.161-0.196 |
| MTT2 (h) | 0.873 (11.0 %) | 0.732-1.043 | 0.731-1.015 |
| ***Interindividual variability (IIV)*** | | |  |
| IIV_KA | 0.779 (50.0 %) | 0.460-1.616 | 0.215-1.462 |
| IIV_VC | 0.047 (34.0 %) | 0.031-0.082 | 0.024-0.070 |
| IIV_F1 | 0.025 (31.0 %) | 0.017–0.042 | 0.013-0.034 |
| IIV_MTT1 | 0.269 (30.0 %) | 0.184-0.433 | 0.137-0.366 |
| IIV_KREN | 0.056 (31.0 %) | 0.037-0.093 | 0.033-0.076 |
| IIV_KM | 0.067 (31.0 %) | 0.045-0.111 | 0.037-0.090 |
| IIV_VM | 0.087 (32.0 %) | 0.059-0.146 | 0.042-0.122 |
| IIV_KME | 0.071(31.0 %) | 0.047-0.117 | 0.039-0.090 |
| IIV_ MTT2 | 0.094 (31.0 %) | 0.065-0.156 | 0.043-0.122 |
| ***Residual unexplained variability (RUV)*** | | |  |
| ***Serum Enalapril*** | | |  |
| Proportional error (σ2) | 0.010 (12.0 %) | 0.008-0.011 | 0.005-0.014 |
| Additive error (ug/l) | 0.186 (23.0 %) | 0.133-0.266 | 0.116-0.425 |
| ***Serum Enalaprilat*** | | |  |
| Proportional error (σ2) | 0.016 (13.0 %) | 0.013-0.019 | 0.010-0.024 |
| Additive error (ug/l) | 0.220 (17.0 %) | 0.170-0.284 | 0.117-0.316 |
| ***Urine Enalapril*** | | |  |
| Proportional error (σ2) | 0.026 (13.0 %) | 0.021-0.032 | 0.011-0.042 |
| ***Urine Enalaprilat*** | | |  |
| Proportional error (σ2) | 0.005 (15.0 %) | 0.004-0.006 | 0.001-0.009 |


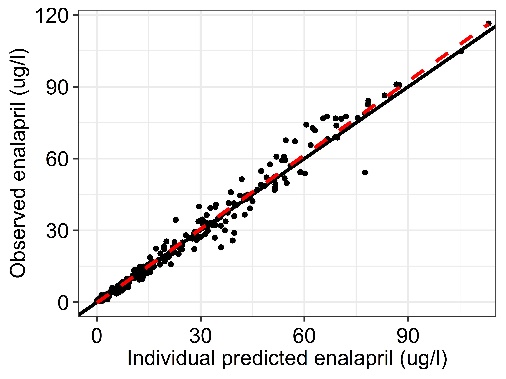

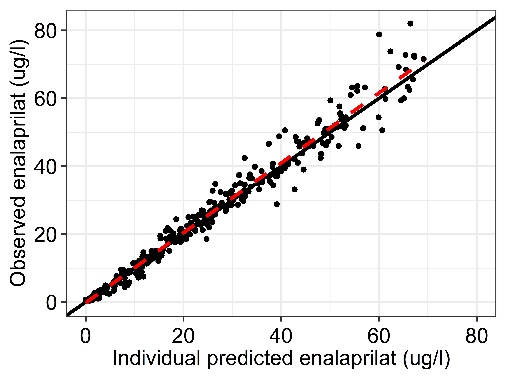

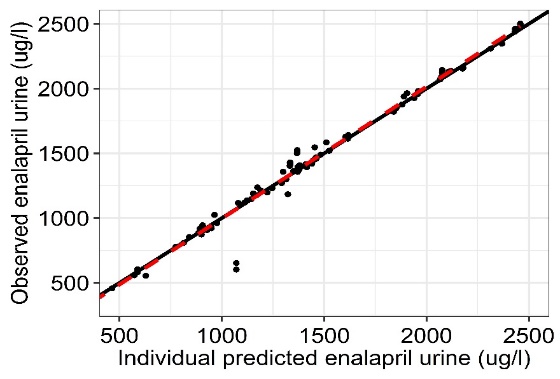

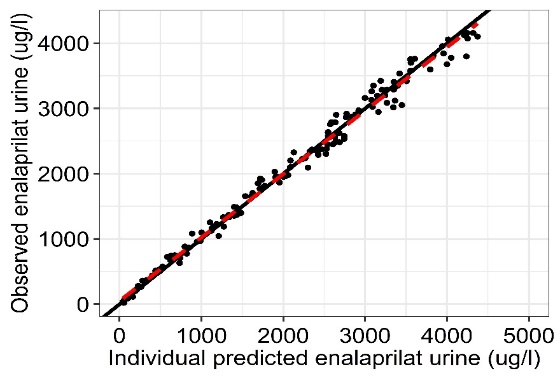

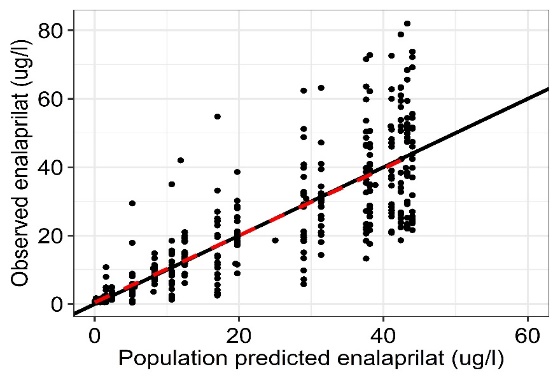

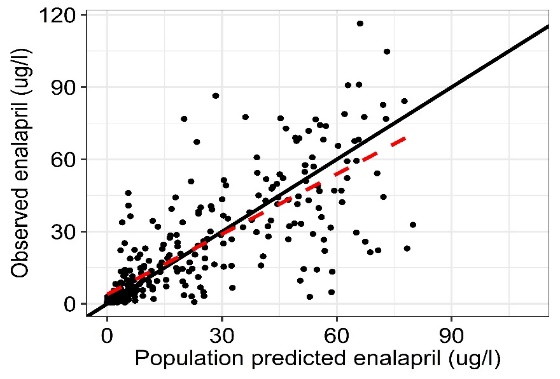

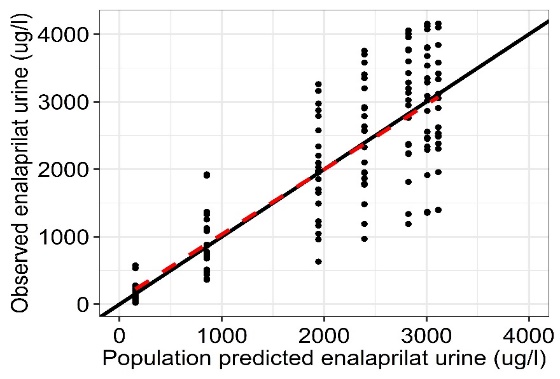

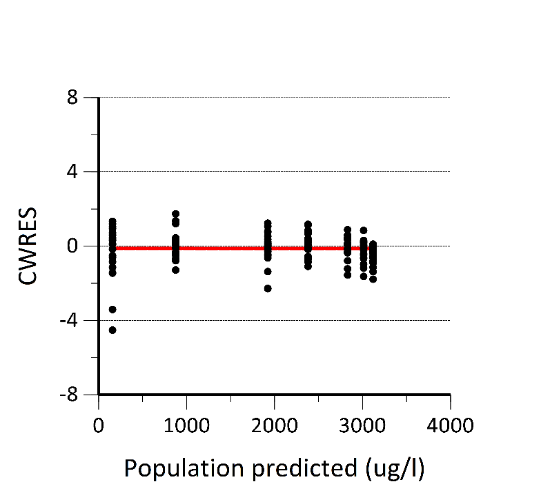

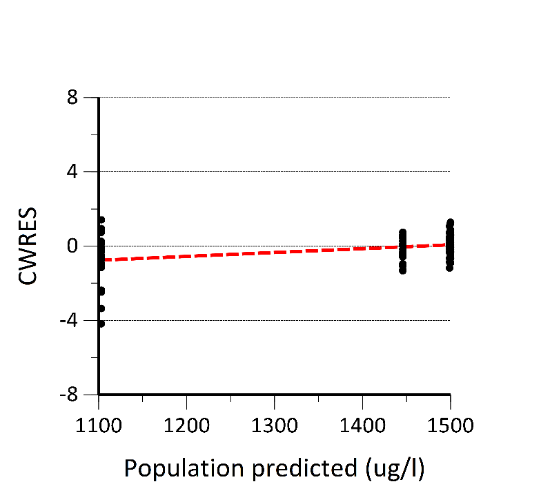

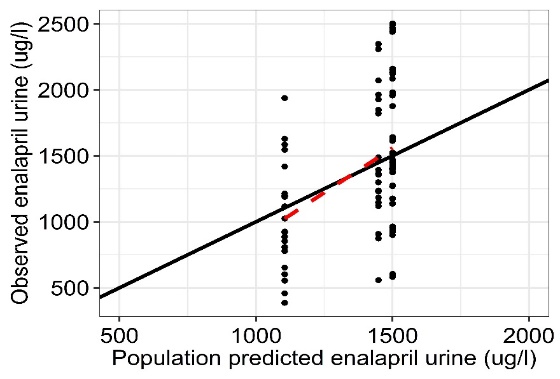

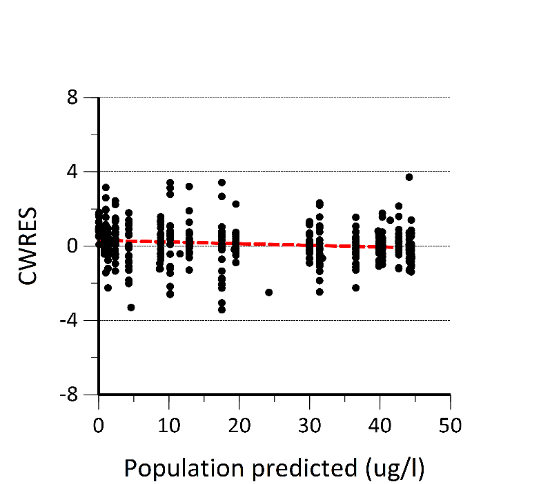

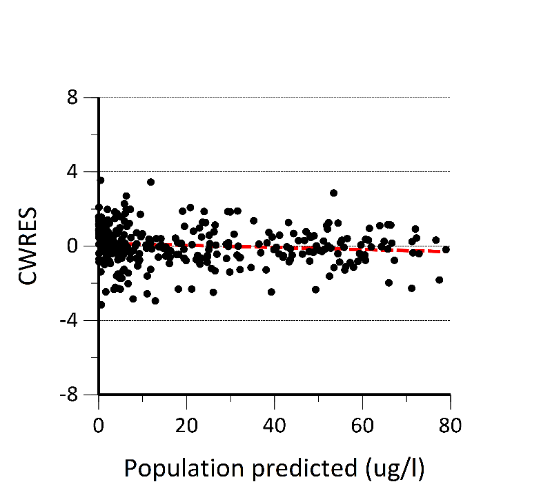


**FIG Supp-1:**


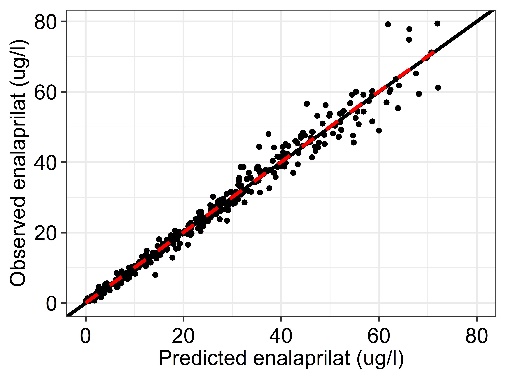

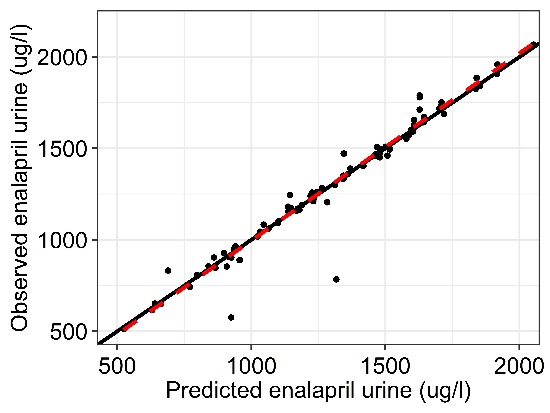

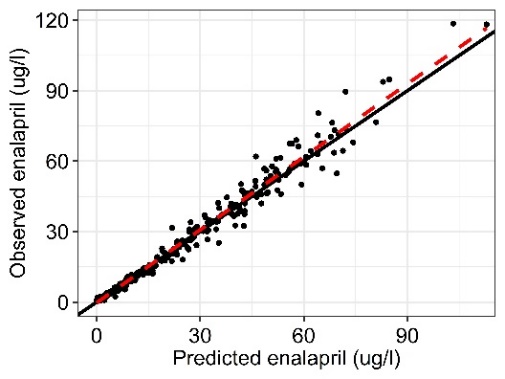

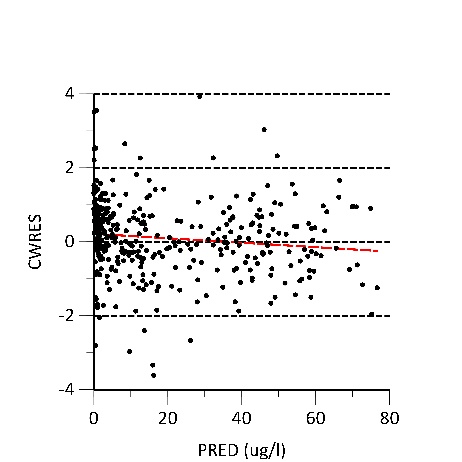

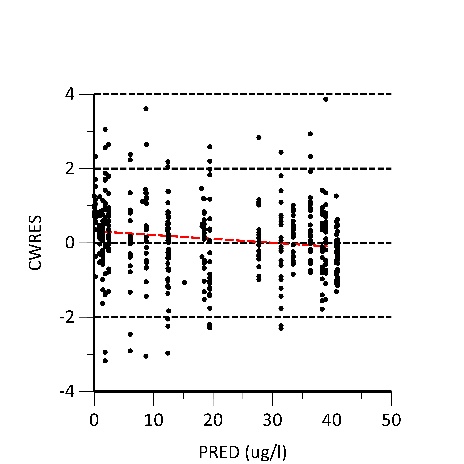

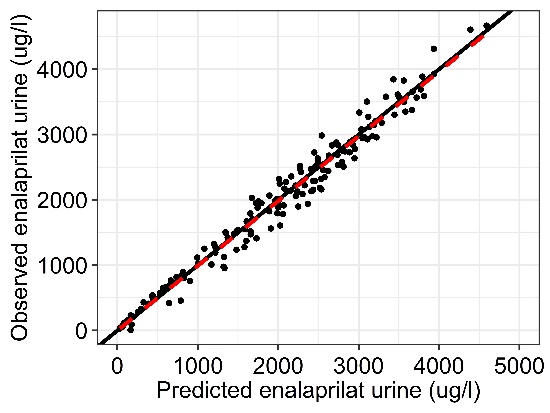

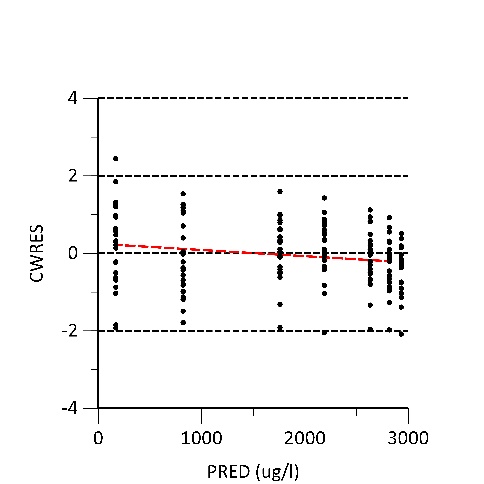

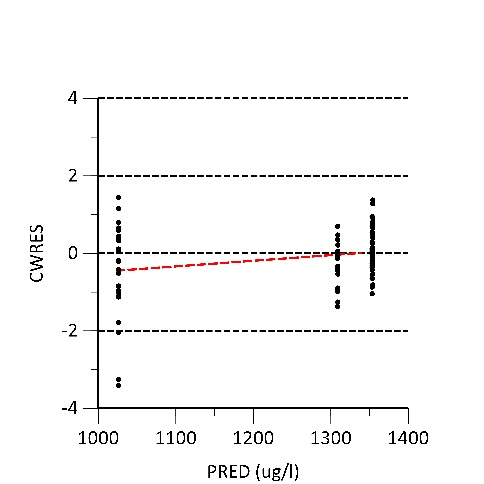

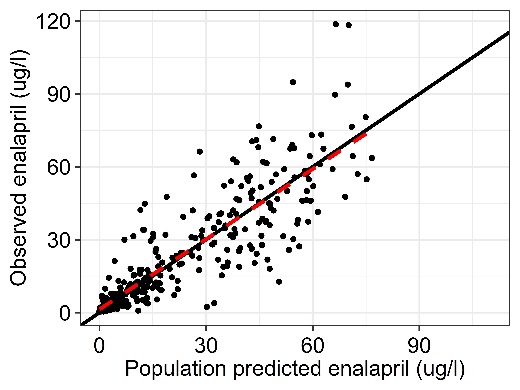

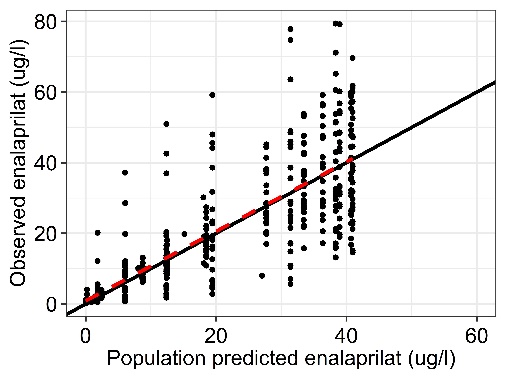

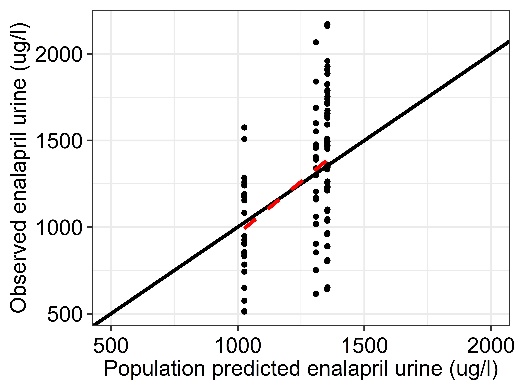

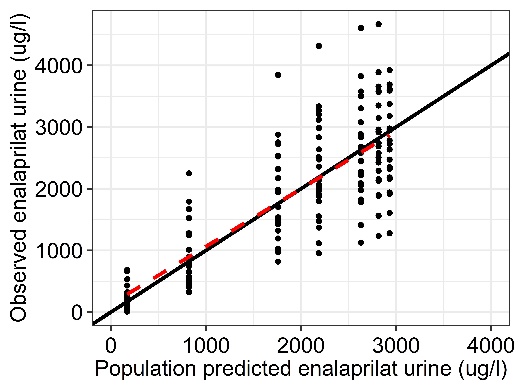


**FIG Supp-2:**
